# Screening of *SOD1*, *FUS* and *TARDBP* genes in patients with amyotrophic lateral sclerosis in central-southern China

**DOI:** 10.1038/srep32478

**Published:** 2016-09-08

**Authors:** Lihua Hou, Bin Jiao, Tingting Xiao, Lu Zhou, Zhifan Zhou, Juan Du, Xinxiang Yan, Junling Wang, Beisha Tang, Lu Shen

**Affiliations:** 1 Department of Neurology, Xiangya Hospital, Central South University, Changsha 410008, China; 2 Hunan Province Key Laboratory in Neurodegenerative Disorders, Central South University, Changsha 410008, China; 3 State Key Laboratory of Medical Genetics, Changsha 410008, China

## Abstract

Amyotrophic lateral sclerosis (ALS) is a fatal neurodegenerative disease affecting motor neurons of the brain, brainstem and spinal cord. To date, mutations in more than 30 genes have been linked to the pathogenesis of ALS. Among them, *SOD1*, *FUS* and *TARDBP* are ranked as the three most common genes associated with ALS. However, no mutation analysis has been reported in central-southern China. In this study, we sequenced *SOD1*, *FUS* and *TARDBP* in a central-southern Chinese cohort of 173 patients with ALS (15 familial ALS and 158 sporadic ALS) to detect mutations. As a result, five missense mutations in *SOD1*, namely, p.D101N, p.D101G, p.C111Y, p.N86S and p.V87A, were identified in three unrelated familial probands and three sporadic cases; two mutations in *FUS* were found in two unrelated familial probands, including an insertion mutation (p.P525_Y526insY) and a missense mutation (p.R521H); no variants of *TARDBP* were observed in patients. Therefore, *SOD1* mutations were present in 20.0% of familial ALS patients and 1.9% of sporadic ALS patients, while *FUS* mutations were responsible for 13.3% of familial ALS cases, and *TARDBP* mutations were rare in either familial or sporadic ALS cases. This study broadens the known mutational spectrum in patients with ALS and further demonstrates the necessity for genetic screening in ALS patients from central-southern China.

Amyotrophic lateral sclerosis (ALS) is a progressive adult-onset disorder that affects upper and lower motor neurons, consequently resulting in muscular weakness and atrophy. Patients eventually die of respiratory failure within 3 to 5 years^1^. It is a clinically heterogeneous disease characterized by different ages of onset, sites of onset, lengths of disease and neurological signs^2^. Further, it has overlapping clinical, genetic and pathological characteristics with frontotemporal dementia (FTD). It is reported that approximately 5–15% patients with ALS meet the criteria for FTD diagnosis. In contrast, clinical features of ALS exist in more than 15% of FTD patients^3^,^4^. The pathogenesis of ALS is complicated, and genetic factors play a relatively important role in patients with ALS. The pattern of inheritance can be autosomal dominant, autosomal recessive, or X-linked^5^. Approximately 5–10% of patients with ALS have a positive family history of the disease.

To date, mutations in more than 30 genes have been linked to the pathogenesis of ALS, but mutations in only a few of them, including *SOD1*, *FUS*, *TARDBP* and *C9orf72*, are present in a significant number of ALS cases^6^,^7^. *SOD1* was the first gene associated with ALS to be identified, and more than 166 mutations have been successively reported^2^, which could account for the occurrence of 20% of familial ALS (fALS) cases and 1–4% of sporadic ALS (sALS) cases^8^. *FUS* mutations have been detected in 4–6% of fALS cases and 0.7–1.8% of sALS cases^9^. Most of these mutations are located in exons 5–6 and exons 14–15 of *FUS*^10^. With regards to *TARDBP,* more than 42 mutations have been found in 5% of fALS cases and 2% of sALS cases^5^. However, there are still no reports of these mutations in central-southern China. Therefore, we performed a comprehensive and systematic screening of *SOD1*, *FUS* and *TARDBP* genes to further analyze the types of mutations and their frequency in patients with ALS from central-southern China. In addition, we further analyzed the clinical characteristics of ALS patients carrying genetic mutations.

## Results

### Genetic Results

Three reported mutations of *SOD1* (p.D101N, p.D101G and p.C111Y) were identified in three unrelated fALS patients, and three reported mutations of *SOD1* (p.N86S, p.V87A and p.C111Y) were identified in three unrelated sALS patients. With regards to *FUS*, one novel mutation (p.P525_Y526insY) and one previously reported mutation (p.R521H) were detected in two unrelated fALS probands. However, no mutations in *TARDBP* were identified. None of these mutations were found in the 500 healthy individuals testd. Genetic results and pathogenic predictions are presented in Table 1. In addition, the mean age of onset for mutation carriers and non-carriers was 44.0 ± 15.2 and 49.0 ± 12.2 years, respectively. The Mann-Whitney U test revealed that there was no significant difference between the groups (*p* = 0.31). No significant differences was found between groups when comparing in male to female ratio (*p* = 0.44).

### *SOD1* mutation in fALS

**Figure 1:**
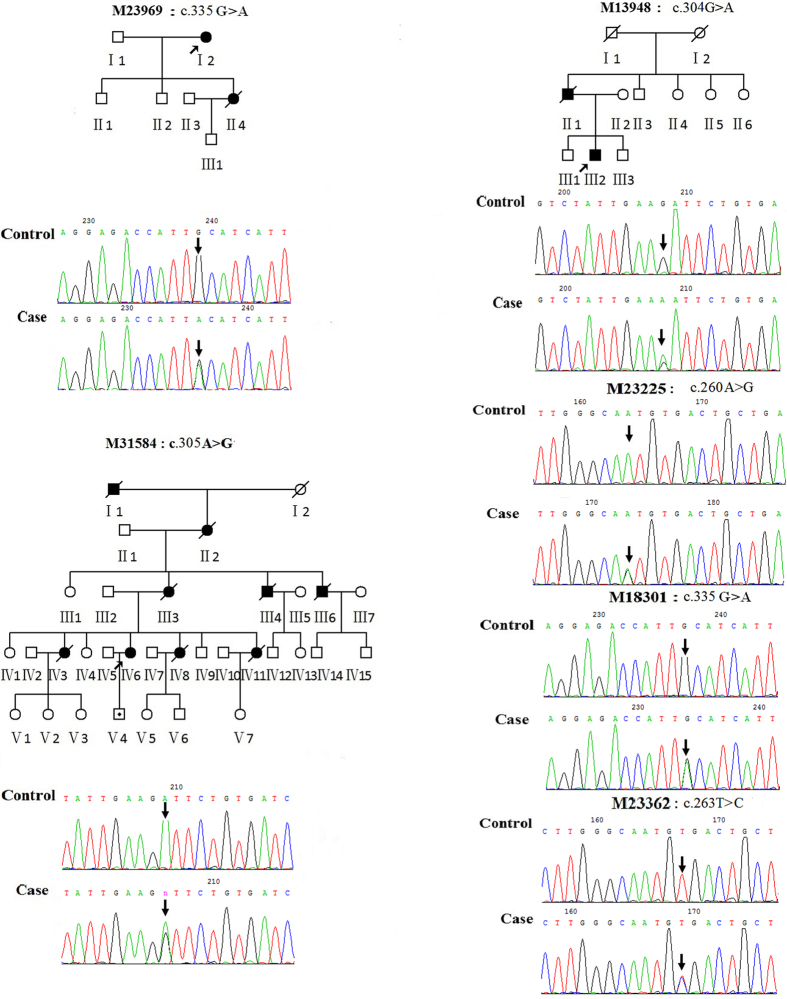
Three pedigrees (M23969, M13948, and M31584) carried *SOD1* mutations, and the corresponding forward sequencing chromatogram of mutations in both probands and controls is shown. The arrow on the pedigree represents the proband. The circles denote females, and squares denote males. Affected individuals are noted by black symbols and unaffected individuals are noted by blank symbols. Carriers are noted by a black dot. Deceased individuals are noted by a slash symbol. M23225, M18301 and M23362 represent forward sequencing chromatogram of sALS patients with *SOD1* mutations. M23969:c.335G>A (p.C111Y); M13948:c.304G>A (p.D101N); M31584:c.305A>G (p.D101G); M23225:c.260A>G (p.N86S); M18301:c.335G>A (p.C111Y); M23362:c.263T>C (p.V87A).

The c.335G>A, p.C111Y mutation was identified in a fALS proband (Fig. 1 M23969: I2). At age 68, she developed a slowly progressive distal weakness in her upper left limb, and one month later, the weakness spread to the lower left limb, while fasciculation and atrophy in the upper left limb were observed. A neurological examination revealed signs of upper motor neuron (UMN) damage, and an electromyogram indicated the presence of generalized chronic progressive neurogenic damage. Her daughter (Fig. 1 M23969: II4) developed progressive weakness in her lower left limb at age 46 and quickly developed weakness, dysarthria and dysphagia in all four extremities within one year. She then died of respiratory failure after18 months. Although her daughter carried the same mutation, she had an earlier age of onset and a seemingly worse clinical phenotype than her mother. Her sons did not carry this mutation or share this clinical phenotype.

The c.304G>A, p.D101N mutation was detected in another fALS patient (Fig. 1 M13948: III2). He began undergoing gradually progressive weakness in his upper right limb at age 26. The distal weakness was predominant, preventing him from performing daily work. One year later, the weakness gradually spread to the lower limbs and upper left limb. Fasciculation and muscle atrophy was observed in all extremities. The tendon reflex was active, the Babinski reflex was detected, and the electromyogram demonstrated chronic reinnervation in all extremities. Two years later, he experienced worsening limb weakness and the development of dysarthria and dysphagia. The patient died of respiratory failure three years after the onset of symptoms. His father (Fig. 1 M13948: II2) began suffering from weakness of the upper limbs at the age of 38. Two years later, his remaining limbs and bulbar muscles were also weakened. Finally, he died of respiratory failure at age 41.

The third *SOD1* mutation c.305A>G, p.D101G was identified in a 47-year-old female who first presented with progressive weakness and systemic fasciculations in her upper left limb (Fig. 1 M31584: IV6). Four months later, she experienced worsening weakness in her upper left limb, and she felt weak in her other limbs. Six months later, dysphagia and dysarthria occurred. Upon admission, a neurologic examination revealed the presence of atrophy, reduced muscle strength, areflexia, and the absence of bilateral Babinski and Hoffman signs in her left hand. The electromyogram revealed extensive chronic neurological changes. Her son was verified as a carrier of the c.305A>G mutation, but he did not present with clinically relevant ALS symptoms (Fig. 1 M31584: V4). Other members of the family experienced a similar, approximately two-year disease course and finally died of respiratory failure in a comparable manner to the proband. Unfortunately, the DNA samples of these family members were unavailable for genetic analysis. Moreover, IV1, IV4 and IV9 did not share this mutation.

### *SOD1* mutations in sALS

Three mutations (p.N86S, p.V87A and p.C111Y) were observed in three unrelated sALS patients. A 58-year-old female patient with the c.260A>G, p.N86S mutation (Fig. 1 M23225) was primarily characterized by progressive weakness and muscle atrophy in her lower limbs beginning at the age of 55. Her tendon reflex was normal and the Babinski reflex was absent. The electromyogram revealed chronic reinnervation in her lower limbs, distal left limb, and paravertebral muscles. The c.335G>A, p.C111Y mutation (Fig. 1 M18301) was identified in a juvenile-onset sALS patient suffering from an aggressive disease progression with muscle atrophy, worsening fasciculation and weakness in all limbs. His deep tendon reflexes were active in all limbs, and the Babinski reflex was present. One year later, he started suffering from occasional choking while drinking and mild dysarthria. The c.263T>C, p.V87A mutation (Fig. 1 M23362) was identified in a sALS patient who developed weakness and muscle atrophy in the upper left limb at 51 years of age. For 24 months, the muscle weakness gradually progressed to the upper right limb and the lower extremities. Physical examinations showed that the deep tendon reflexes of all limbs were brisk and the Hoffman sign was present. Electromyography revealed extensive chronic progressive neurological damage in all four extremities.

### *FUS* mutations

**Figure 2:**
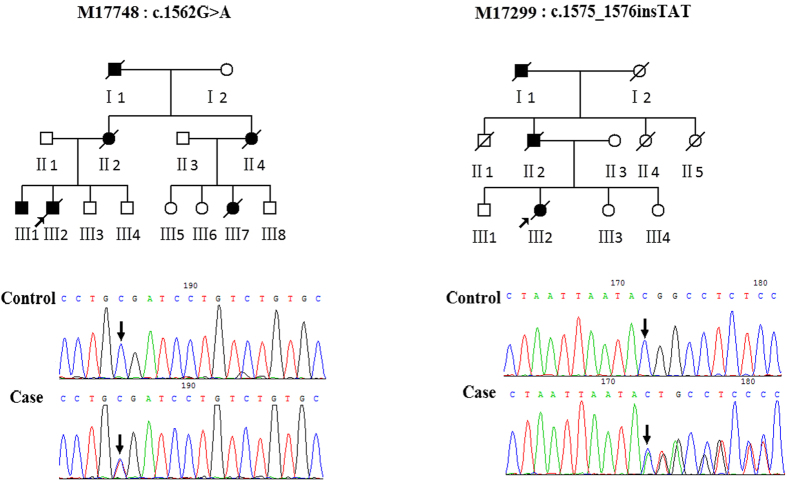
Two corresponding reverse sequencing chromatograms depicting *FUS* mutations in ALS patients and controls. M17748:c.1562G>A (p.R521H); M17299:c.1575_1576insTAT (p.P525_Y526insY).

The c.1562G>A, p.R521H mutation (Fig. 2 M17748: III:2) was detected in a fALS patient who initially suffered from progressive weakness in the upper right limb at age 41. One year later, the weakness, fasciculation, and muscle atrophy progressed to other limbs. His symptoms were primarily characterized by proximal muscle weakness without signs of upper motor neuron damage or cognitive impairment. The electromyogram showed spontaneous denervation activity in all limbs. He presented with dysarthria and dysphagia after 13 months and died at age 44. His brother, who had the same mutation, also had a similar clinical phenotype and disease duration. III:3 and III:4 did not share this mutation. Therefore, the c.1562G>A, p.R521H mutation completely co-segregated with the disease phenotype within this family.

The novel mutation c.1575_1576insTAT, p.P525_Y526insY was identified in a female proband (Fig. 2 M17299: III2). Her initial symptoms, beginning at age 43, were progressive weakness of the upper left limb and slow progression of weakness into the upper right limb and lower limbs. Meanwhile, muscle atrophy of the upper left limb occurred. Due to the proximal weakness, she could not lift her arms or go down stairs. A neurological examination showed that the tendon reflexes in her upper limbs were disappearing, while the tendon reflexes in her lower limbs were pathologically brisk. The electromyogram showed general spontaneous denervation activity and a loss of motor units in the tibialis anterior. Approximately 28 months later, the weakness in her limbs, mild dysarthria and dysphagia worsened, and she finally died of respiratory failure. Her father presented with a similar clinical phenotype and disease duration. Unluckily, his DNA sample was not obtained. III:1, III:3, and III:4 did not carry this mutation and share this clinical phenotype.

## Discussion

In this study, we systematically described the frequencies of *SOD1*, *FUS* and *TARDBP* mutations in patients with ALS from central southern China. Mutations in *SOD1* were present in 20.0% of fALS patients and 1.9% of sALS patients, which is consistent with previous studies in western populations^8^, and indicates that *SOD1* mutations play a key role in the pathology of ALS patients from different ethnicities. In addition, the frequency of *FUS* mutation was approximately 13.3% in fALS patients, which is consistent with reports from several other populations^11^,^12^,^13^,^14^, but is higher than the frequencies found in Catalan (8%)^15^, German (2.4–6.9%)^16^,^17^, Italian (4.4%)^12^, and Belgian (2.9%)^18^ populations. This difference may be due to small sample sizes and the differing ethnicities of fALS patients. Although *FUS* mutations were not found in sALS patients, this is probably due to both the selective detection of exons in mutational hotspot regions and the use of small sample sizes. Zou ZY reported that *FUS* mutations could be present in approximately 1.0% of northern Chinese sALS patients, which is similar to the 1.6% mutation frequency found in Korean patients and the 1.25% mutation frequency found Italian patients^12^,^14^,^19^,^20^. With regards to the frequency of *TARDBP* mutation, it appears that the frequency of *TARDBP* mutations in Caucasian populations (Italy: 2.7%; France and Quebec: 4.5%) was higher than that in Asian populations^21^,^22^,^23^,^24^. The prevalence was different, even within the same population. Zou ZY reported that *TARDBP* mutations were only present in 0.73% of northern Chinese sALS patients^25^, but Soong’s research showed that *TARDBP* mutations have a higher frequency (4.3%), second only to *SOD1* mutations (7.5%), in Taiwanese ALS patients, especially fALS patients^26^. Undeniably, our study did not identify *TARDBP* mutations, which is probably due to our small sample sizes and the ethnic differences of our study population. In addition, we previously reported that *C9orf72* expansion was present in approximately 8.33% of fALS patients, which was lower than the frequency of *FUS* and *SOD1* mutation^27^. This study indicates that in Chinese ALS patients, we should screen *SOD1* mutations first, then *FUS* mutations, and then *C9orf72* and *TARDBP* mutations.

In current study, four mutations of *SOD1* (p.N86S, p.V87A, p.D101N, p.D101G) were identified firstly in Chinese ALS patients. p.N86S mutation was previously first identified in a juvenile onset case of fALS, all reported affected family members present worse phenotype and ephemeral duration^28^, while in our study, the sALS patients with this same mutation showed only mild clinical phenotypes. Although Andersen had previously reported the p.V87A mutation, the clinical features of the patients were not detailed^29^. It was noted that the patients with the p.V87A mutation were prone to experience relatively typical ALS-related clinical symptoms. The third mutation we identified, p.D101N, has been associated with a rapid disease course, with patients exhibiting a low propensity towards aggregate formation. In our study, the patients with the D101N mutation presented a similar clinical phenotype, with a relatively early age of onset and rapid ALS disease progression^30^. The fourth mutation we identified, p.D101G, is located in same site as p.D101N and was first detected in an ALS family from the UK^31^. In our study, all patients with the D101G mutation experienced a short disease duration. Both the D101N and D101G mutations have been shown to be associated with a rapidly progressing disease lasting approximately 2.5 years. This rapid disease progression may be caused by mutant *SOD1* aggregation^30^,^32^,^33^. The final mutation we identified, p.C111Y, has been identified in North American, Japanese and Chinese families. The most common initial symptoms were primarily located in the spinal cord, and the disease progressed relatively slowly^34^,^35^. According to our results, all five of these mutations were located in exon 4, which appears to be a hotspot of variation in comparison to other exons.

With regards to *FUS* mutations, to our knowledge, the p.P525_Y526insY mutation is the first reported in an ALS patient. This discovery broadens the spectrum of known genetic mutations in patients with ALS. The p.P525_Y526insY mutation is located in an Arg/Gly-rich region of the C-terminal domain of *FUS* that contains the nuclear localization signal (NLS)^36^. C-terminal fALS-associated *FUS* mutations affect the major NLS of the protein and thus impair its nuclear import^37^. The P525L mutation at the same site has shown the strongest degree of cytosolic mislocalization and was reported to cause especially aggressive forms of fALS^38^,^39^. Thus, it is possible that the p.P525_Y526insY mutation disrupts nuclear import. The previously reported p.R521H mutation, located in a mutational hotspot region of the *FUS* gene, may result in aberrant localization and cytoplasmic accumulation of the mutant FUS protein^40^,^41^. Patients with the p.R521H mutation were prone to notice spinal symptoms first. Moreover, the p.R521H mutation was shown to correlate with a longer disease course^42^. In fact, several *FUS* mutations cause disease characteristics distinctive of ALS patients. The p.P525L variant, frameshift mutations and gene deletions were identified to associate with juvenile disease onset and aggressive disease duration^2^,^43^. The p.R521C mutation were particularly associated with phenotypes of neck and proximal muscle weakness^44^,^45^.

In summary, this study not only verified the high frequency of *SOD1* mutations in Chinese fALS or sALS patient groups, but it also highlighted the importance of *FUS* mutations in Chinese fALS groups and revealed that *TARDBP* mutations are an uncommon cause of sALS or fALS in Chinese populations. In addition, we identified one novel mutation and broadened the spectrum of known *FUS* mutations contributing to ALS. Meanwhile, we provided a basis for the further study of genotype-phenotype correlations.

## Methods

### Subjects

A total of 173 unrelated patients with ALS were recruited from the Department of Neurology, Xiangya Hospital. All patients were of Han nationality, and were from the central southern China region, including the provinces of Hunan, Hubei, Jiangxi, Henan, and Guangxi. All patients, including 158 sALS patients (male: 69.4%; age at onset: 49.4 ± 12.1 years) and 15 fALS patients (male: 66.7%; age of onset: 47.5 ± 12.2 years), met the EI Escorial criteria for ALS diagnosis^46^. The 500 healthy individuals that were matched by gender, age and geographic region (male: 67.0%; age at onset: 48.5 ± 12.5 years). They were from the Xiangya Hospital Health Center. This study was approved by the Ethics Committee of Xiangya Hospital of the Central South University in China (equivalent to an Institutional Review Board) and carried out in accordance with the approved guidelines. Written informed consent was obtained from all participants.

### Mutation screening

Genomic DNA was extracted from peripheral blood leukocytes using standard methods. The quality and quantity of DNA were assessed with a fluorometer. All DNA samples were diluted to 50 ng/uL. Using Sanger sequencing, all patient DNA was screened for mutations in all exons of *SOD1* (NM_000454.4) and *TARDBP* (NM_007375.3) and in exons 5–6 and exons 14–15 of FUS (NM_004960.3). All primers were designed by Primer 5 software to amplify coding regions of *SOD1*, *TARDBP* and *FUS* using polymerase chain reaction (PCR) and to flank non-coding regions. Primers and PCR reaction conditions are listed in Supplementary Data 1. PCR products were sequenced using identical forward and reverse primers with BigDye terminator v3.1 sequencing chemistry on an ABI 3730xl DNA analyzer (Applied Biosystems). The DNA sequences were analyzed using Sequencher software, version 4.2. When a novel mutation was found, we first confirmed whether it was a novel mutation using the HGMD database (http://www.hgmd.cf.ac.uk/ac) and the dbSNP database (http://www.ncbi.nlm.nih.gov/snp). We then screened for the presence of the novel mutations in healthy controls. Finally, we used SIFT online software to predict the pathogenicity of the novel variants.

### Statistical analysis

Statistical analysis of clinical data was performed using SPSS 18.0 (SPSS, Inc., Chicago, IL). To compare the differences among the two groups, the Mann-Whitney U test was used for continuous variables and Chi-square test was used for categorical variables. The threshold of statistical significance was set at *p* < 0.05.

## Additional Information

**How to cite this article**: Hou, L. *et al.* Screening of *SOD1*, *FUS* and *TARDBP* genes in patients with amyotrophic lateral sclerosis in central-southern China. *Sci. Rep.* **6**, 32478; doi: 10.1038/srep32478 (2016).

## Supplementary Material

Supplementary Information

## Figures and Tables

**Table 1:** The mutations on *SOD1* and *FUS* gene found in this study.

N0.	Gene	Inheritance	Location	Position	cDNA	Protein	Mutation type	Value	Reference/Novel
M13948	*SOD1*	F	Exon4	33039635	c.304G>A	p.D101N	Missense	0.001 damaging	Jones (1994)
M23969	*SOD1*	F	Exon4	33039666	c.335G>A	p.C111Y	Missense	0.000 damaging	Eisen^34^
M31584	*SOD1*	F	Exon4	33039667	c.305A>G	p.D101G	Missense	0.000 damaging	Yulug^31^
M23225	*SOD1*	S	Exon4	33039591	c.260A>G	p.N86S	Missense	0.000 damaging	Hayward^28^
M23362	*SOD1*	S	Exon4	33039594	c.263T>C	p.V87A	Missense	0.000 damaging	Andersen^29^
M18301	*SOD1*	S	Exon 4	33039666	c.335G>A	p.C111Y	Missense	0.000 damaging	Eisen^34^
M17748	*FUS*	F	Exon 15	31202740	c.1562G>A	p.R521H	Missense	0.000 damaging	Kwiatkowski^39^,^41^
M17299	*FUS*	F	Exon15	31202753	c.1575_1576insTAT	p.P525_Y526insY	Insertion	NA	In this study
