# Supplementary material for: Screening of SOD1, FUS and TARDBP genes in patients with amyotrophic lateral sclerosis in central-southern China
Source: Sci Rep. 2016 Sep 8;6:32478. doi: 10.1038/srep32478 (PMC5015023; doi:10.1038/srep32478)
Supplement: Supplementary Information [file srep32478-s1.doc]

**Screening of *SOD1*, *FUS* and *TARDBP* genes in patients with** **amyotrophic lateral sclerosis in** **central-southern China**

Lihua Hou ^1,3^, Bin Jiao ^1,3^, Tingting Xiao ^1,3^, Lu Zhou^1,3^,Zhifan Zhou ^1,3^, Juan Du ^1,3^,Xinxiang Yan ^1,3^, Junling Wang ^1,3^, Beisha Tang ^1,2,3^, Lu Shen ^1,2,3*^

1. Department of Neurology, Xiangya Hospital, Central South University, Changsha 410008, China

2. State Key Laboratory of Medical Genetics, Changsha 410008, China

3. Hunan Province Key Laboratory in Neurodegenerative Disorders, Central South University, Changsha 410008, China

* Corresponding to Dr. Shen,

Department of Neurology, Xiangya Hospital, Central South University, #87 Xiangya Road, Changsha 410008, China. Phone: +86-731-84327623 Fax: +86-731-84327332. E-mail address: shenlu2505@126.com.

**Supplementary materials**

**Table 1:** **PCR conditions and primer sequences of *SOD1***.

| ***SOD1* exon** | **Primer sequence** | **Size in bp** | **T_m_ in ℃** |
| --- | --- | --- | --- |
| Exon1 | F: GGGATTGAGGTGTAGCGACTT  R: CCTCGCAAACAAGCCTCC | 663 | 58.0 |
| Exon2 | F: CCATCTCCCTTTTGAGGACA  R: GGGGCTACTCTACTGTTTAT | 369 | Touch down |
| Exon3 | F:TCCATGGGAAGTTTTAGCAG  R: CAGGGGTTTAGATGAGTCAG | 301 | Touch down |
| Exon4 | F: TAGTGTGTAGACGTGAAGCC  R: CTAACAATCAAAGTGAAAAG | 355 | Touch down |
| Exon5 | F: AAACTGCCAAAGTAAGAG  R: TAAGTGCCATACAGGGTT | 766 | 62.2 |

PCR reaction conditions of exons 2, 3, and 4: (95℃ 30s- annealing at 66.4℃ and dropping 0.5℃for each cycle 30s-72℃32s) *12cycles-（95℃ 30s -58.0℃ 30s- 72℃ 32s）*25cycles-72℃ 10min.

PCR reaction conditions of exon 1: 95℃ 5min-(94℃ 30s- annealing 58.0℃ 30s-72℃30s)*32cycles-72℃ 10min.

PCR reaction conditions of exon 5: 95℃ 5min-(94℃ 30s- annealing 62.2℃ 30s-72℃30s)*32cycles-72℃ 10min.

**Table 2: PCR conditions and primer sequences of *TARDBP***

| ***TARDBP* exon** | **Primer sequence** | **Size in bp** | **T_m_ in ℃** |
| --- | --- | --- | --- |
| Exon2 | F: ACCCTTACCTTCACCTCGTCA  R:TTCAGGAGACATTCTGCCACC | 519 | Touch down |
| Exon3 | F: CAGAAAAGCACCTCAGACA  R: GTTTGACGCCAGAACCTA | 474 | Touch down |
| Exon4 | F: CCACTGCATCCAGTTGAAACCAT  R: AACACACCCTGCCGCTATCTTTT | 354 | 55.4 |
| Exon5 | F: CACTGCTATCCAAGGCGAAT  R: CTCTGTCACTGGGCTGGAAT | 386 | Touch down |
| Exon6 | F:CCTCTGGCTTTAGATAAATTAATGCTTG  R: ATTTGAATTCCCACCATTCTATACC | 684 | Touch down |

PCR reaction conditions of exons 2, 3, 5, and 6: 95℃ 5min-(94℃ 30s- annealing at 62.0℃ and dropping 0.5℃for each cycle 1min -72℃ 1min) *15cycles-（94℃ 30s -56℃ 1min- 72℃ 1min）*28cycles-72℃ 10min.

PCR reaction conditions of exon 4: 95℃ 5min-(94℃ 30s- annealing 55.4℃ 30s-72℃30s) *35cycles-72℃ 10min.

**Table 3: PCR conditions and primer sequences of *FUS***

| ***FUS* exon** | **Primer sequence** | **Size in bp** | **T_m_ in ℃** |
| --- | --- | --- | --- |
| Exon5 | F: TGTTGGGTACAGAGAATGGAC  R: AGCCTCAGCAACAGAGACAG | 375 | Touch down |
| Exon6 | F: GGCACTTGTCAAACCTTTTC  R: CACTCCCCACCAAAGATACT | 370 | Touch down |
| Exon14 | F: CACATGGGTAAGAAAGGCAG  R: TCTCAACAAAACCCTGTTATCC | 348 | Touch down |
| Exon15 | F:TACTCGCTGGGTTAGGTAGG  R:TTCCAGGAAAGTGAAAGGG | 344 | Touch down |

PCR reaction conditions: 95℃ 5min-(94℃ 30s-annealing at 62℃ and dropping 0.5℃for each cycle 1min-72℃ 1min) *15cycles-（94℃ 30s -56℃ 1min- 72℃ 1min）*28cycles-72℃ 10min.
